# Characterizing hub biomarkers for metabolic-induced endothelial dysfunction and unveiling their regulatory roles in EndMT through RNA sequencing and machine learning approaches

**DOI:** 10.3389/fcvm.2025.1585030

**Published:** 2025-05-15

**Authors:** Qi Sun, Longchuan Xie, He An, Wei Chen, Qirong Yang, Peng Wang, Yijun Tang, Chunyan Peng

**Affiliations:** ^1^ Clinical Molecular Diagnostic Center, Taihe Hospital, Hubei University of Medicine, Shiyan, Hubei, China; ^2^ Hubei Key Laboratory of Embryonic Stem Cell Research, Hubei University of Medicine, Shiyan, Hubei, China

**Keywords:** cardiovascular disease, endothelial dysfunction, lipid metabolism, EndMT, hub genes, bioinformatics analysis, machine learning, ceRNA network

## Abstract

**Background:**

Metabolic disorder and endothelial dysfunction (ED) are key events in the development and pathophysiology of atherosclerosis and are associated with an elevated risk of Cardiovascular disease (CVD). The pathophysiology remains incompletely understood.

**Methods:**

Leftover serum samples were collected and stored at −20 °C until study. Serum specimens were mixed to obtain pooled high glucose serum (GLU group) (11.97 ± 2.09 mmol/L); pooled elevated low-density lipoprotein serum (LDL group) [3.465 (3.3275, 3.6425 mmol/L)]; pooled high triglycerides serum (1.15 ± 0.35 mmol/L) (TG group); Subsequently, Human umbilical vein endothelial cells (HUVECs) were exposed to culture media supplemented with these pooled serum or control serum for 72 h. Whole transcriptome sequencing was performed to characterize gene expression profiles and data were analyzed using GSEA, GO, KEGG. qPCR was used to validate the gene expression.

**Results:**

A total of 306 mRNAs and 523 lncRNAs were identified as differentially expressed in the GLU group, 335 mRNAs and 471 lncRNAs in the LDL group, and 364 mRNAs and 562 lncRNAs in the TG group, compared to the control group. These genes are primarily involved in inflammation, lipid metabolism, and EndMT pathways. By integrating differentially expressed mRNA and curated EndMT-related gene sets from the KEGG, GO, and dbEMT2.0 databases, we identified 52 differentially expressed genes associated with EndMT under metabolic stress conditions. Utilizing machine learning techniques, we established an EndMT-associated gene diagnostic signature comprising CD36, ISG15, HSPB2, and IRS2 for the diagnosis of AS, which achieved an AUC of 0.997. The model was subsequently validated across three independent external cohorts (GSE43292, GSE28829, GSE163154), in which it consistently demonstrated strong diagnostic performance, with AUC values of 0.958, 0.808, and 0.884, respectively. The ceRNA networks associated with EndMT are constructed and related lncRNAs including LINC002381, VIM-AS1, and ELF-AS1 were significantly upregulated in peripheral blood samples.

**Conclusions:**

This study identified novel biomarkers for ED. These findings may provide both a potential biomarker and therapeutic target for the prevention and treatment of atherosclerosis and CAD.

## 1 Introduction

Cardiovascular disease (CVD) represents a significant global health concern, with mortality increasing from approximately 12.1 million in 1990 to nearly 20.5 million in 2021 (1). The pathogenesis and development of CVD are closely related to a series of metabolic disorders, including diabetes, obesity, hypertension, and dyslipidemia. With the aging population and the increasing prevalence of metabolic disorders, the global burden of CVD continues to increase (2). This fact highlights the pressing necessity to comprehend how it originates, make progress in its control, and explore therapeutic possibilities for reducing its impact (3).

Endothelial cells (ECs) form the inner lining of blood vessels that is crucial for vascular function and homeostasis. They regulate vascular tone, oxidative stress, and permeability. Endothelial Dysfunction (ED) leads to increased permeability, leukocyte adhesion, and thrombosis (4). During the inflammatory process induced by different risk factors as hypertension, oxidized low-density lipoprotein (oxLDL) and diabetes, there is an increase in the production of interleukin-1 (IL-1), interleukin-6 (IL-6), tumor necrosis factor-α (TNF-α) and C-reactive protein (CRP) that generate the endothelial proinflammatory phenotype characterized by an increase in E-selectin, vascular cell adhesion molecule-1 (VCAM-1) and intercellular adhesion molecule 1 (ICAM-1) expression (5). Therefore, there is a greater interest in the search for new biomarkers and therapeutic strategies that help to prevent ED and reduce the risk of developing CVD and its complication (6).

In the previous studies, culture medium with glucose or ox-LDL were used to treat ECs to establish ED *in vitro* model, however, under physiological conditions, circulating glucose, lipids, hormones, inflammatory mediators, anticoagulant and procoagulant factors were all contributed to dynamic equilibrium of ECs (7, 8). Conventional single-component culture systems employing supraphysiological glucose concentrations induce cellular glucose desensitization (9) and may introduce potential biological artifacts, which limit the accuracy of modeling physiological conditions. This methodological limitation, coupled with the absence of multifactorial *in vitro* models that can incorporate clinically relevant parameters has limited hypothesis-driven studies and, in turn, limited our progress in understanding ED mechanisms. Treatment with pooled clinical serum with altered metabolic parameters could better mirrors the microenvironment reality may be better model for ED study. In this study, pooled serum (high-glucose; high low-density lipoprotein; high triglycerides) from individuals were used to treat Human umbilical vein endothelial cells (HUVECs). Subsequently, whole-transcriptome sequencing was employed to determine the expression profiles of mRNAs and key noncoding RNAs (including circRNAs and lncRNAs) within these models, with the intention of exploring their potential regulatory mechanisms.

## 2 Materials and methods

### 2.1 Subject

Individuals who underwent physical examination at the Health Management Center of Taihe Hospital from January to March 2021 were included as research subjects in this study. Fasting venous blood samples were collected from these participants for routine blood tests and biochemical analyses at the hospital laboratory department, using accredited laboratory methods. The hematological and biochemical investigations which were performed included hemoglobin, total leukocyte count, platelet count, neutrophil level, blood sugar, urea, creatinine, sodium, potassium, serum total cholesterol (TC), serum triglycerides (TG), and serum low-density lipoprotein (LDL). A residual serum volume of 2–5 ml was separated from the remaining blood samples by centrifugation at 1,500 rpm for 15 min at 4 ℃ and stored at −80 ℃ for future use. All subjects had no history of severe preexisting infections, immunological or cardiovascular diseases that required long-term medication, hypertension, diabetes, and chronic kidney disease. This study received approval from the Ethics Committee of Hubei University of Medicine.

### 2.2 Preparation of pooling serum

Matching serum samples from healthy individuals (*n* = 80) were selected based on sex, age, and test results. Serum from 20 samples exhibiting isolated elevations in blood glucose levels (11.97 ± 2.09 mmol/L, GLU group) was pooled by combining 3 ml from each sample, yielding a total volume of 60 ml of high-glucose pooled serum with a final concentration of 12.36 mmol/L. Likewise, serum from 20 samples showing isolated elevations in LDL levels [3.465 (3.3275, 3.6425 mmol/L), LDL group] was pooled in the same manner, resulting in a total volume of 60 ml of high-LDL pooled serum (final concentration: 3.30 mmol/L). Serum from 20 samples with isolated elevations in TG levels [3.725 (3.0125, 5.08) mmol/L, TG group] was pooled, producing a total volume of 60 ml of TG pooled serum (final concentration: 3.40 mmol/L). All other measured indicators remained within the normal range for each group (Supplementary Table 1). Additionally, twenty normal serum samples, each with all indicators within normal ranges, were pooled to serve as control samples (Con group). All serum samples were maintained at 4 °C throughout the pooling process, and the pooled serum was stored at −80 °C for subsequent experiments. Biochemical indices of pooled samples were measured and are reported in Table 1.

**Table 1:** Baseline biochemical characteristics of pooled serum samples.

Biochemical parameter (unit)	Pooled high GLU	Pooled high LDL	Pooled high TG	Pooled control
TC (mmol/L)	4.22	**6**.**73**	4.12	4.34
TG (mmol/L)	1.05	1.28	**3**.**40**	1.04
HDL-C (mmol/L)	1.15	1.50	1.01	1.26
LDL-C (mmol/L)	1.90	**3**.**30**	1.66	1.92
Glucose (mmol/L)	**12**.**36**	4.69	4.99	4.9
Apolipoprotein A1 (g/L)	0.98	1.06	0.98	1.03
Apolipoprotein B (g/L)	0.78	1.11	0.72	0.75
Lipoprotein (a) (mg/L)	83.74	182.15	93.66	57.72

Reference values: Total Cholesterol (TC, 2.8–5.68 mmol/L), Triglycerides (TG, 0.28–1.8 mmol/L), High-Density Lipoprotein Cholesterol (HDL-C, 0.9–1.6 mmol/L), Low-Density Lipoprotein Cholesterol (LDL-C, 1.5–3.11 mmol/L), Glucose (3.9–6.1 mmol/L), Apolipoprotein A1 (ApoA1, 1.06–1.8 g/L), Apolipoprotein B (ApoB, 0.6–1.14 g/L), Lipoprotein(a) (Lp(a), 0–300 mg/L). Bold values indicate measurements outside the normal reference range.

### 2.3 Human umbilical vein endothelial cells culture and treatments

Human umbilical vein endothelial cells (HUVECs) were isolated from umbilical cord veins obtained from the Obstetrics and Gynecology department of Taihe Hospital. The HUVECs were cultured in Dulbecco's modified Eagle's medium, supplemented with 10% fetal bovine serum, 100 U/ml penicillin, and 100 mg/ml streptomycin. The cultures were maintained at 37 °C in a 5% CO_2_ environment. ECs ranging from passages three to five were utilized in the experiments. HUVECs were seeded at a density of 15,000 cells/cm^2^, and on the following day, the culture medium was replaced with fresh medium containing a Penicillin–Streptomycin solution at a final concentration of 1% and 15% v/v of pooled serum from the GLU, LDL, TG, and Con groups. The cells were treated for a duration of 72 h.

### 2.4 RNA extraction, cDNA libraries preparation, and high-throughput RNA sequencing

Each treatment group consisted of four independent biological replicates (*n* = 4 per group). In total, 16 samples were sent to Oebiotech Corporation (Shanghai, China) for RNA sequencing. Total RNA was extracted using the mirVana miRNA Isolation Kit (Ambion) in accordance with the manufacturer's protocol. The integrity of the RNA was assessed using the Agilent 2100 Bioanalyzer (Agilent Technologies, Santa Clara, CA, USA). Only samples with an RNA Integrity Number (RIN) ≥7 were selected for further analysis. Libraries were constructed using TruSeq Stranded Total RNA with Ribo-Zero Gold, following the manufacturer's instructions. These libraries were subsequently sequenced on the Illumina HiSeq™ 2500 sequencing platform, resulting in 150 bp paired-end reads.

### 2.5 Identification and characterization of DElncRNAs and DEmRNAs

The long non-coding RNA (lncRNA) profiles were identified by screening the merged transcript sets based on five criteria: (1) transcript length of at least 200 nucleotides; (2) transcripts containing two or more exons; (3) exclusion of transcripts overlapping with exon regions annotated as coding genes in the database; (4) transcripts with an FPKM (fragments per kilobase of exons per million mapped fragments) of at least 0.1 in one or more groups; (5) identification of transcripts with low protein-coding potential using four distinct algorithms (CNCI v1.0, PLEK v1.2, CPC2-beta, and Pfam v30). Each transcript was required to have a minimum of 0.1 million mapped fragments in one or more groups. We utilized the DESeq2 R package for RNA-seq data normalization and processing, and applied it to each subset to analyze differentially expressed mRNAs (DEmRNAs) and long non-coding RNAs (DElncRNAs) from our sequencing results. DEmRNAs were identified using the criteria of absolute log2-fold change (|log2FC|) ≥ 0.5 and *P* < 0.05. Similarly, lncRNA transcripts with |log2FC| ≥ 1 and *P* < 0.05 were considered differentially expressed.

### 2.6 Functional analyses of DEmRNAs and DElncRNAs

The Metascape database (https://metascape.org/gp/) was selected for the enrichment analysis of overlapping DEmRNAs, with the results visualized in enriched bar graphs. Annotation and visualization were conducted for Gene Ontology (GO) and Kyoto Encyclopedia of Genes and Genomes (KEGG) pathway enrichment analyses to investigate the potential roles and functions of the identified DEmRNAs. The GO annotation analysis, based on the GO database, was performed for three ontologies: biological process (BP), molecular function (MF), and cellular component (CC). Additionally, Gene Set Enrichment Analysis (GSEA) was employed to elucidate the biological significance of characteristic genes. The R package clusterProfiler can be utilized to perform KEGG, GO, and GSEA enrichment analyses. To explore the potential functions of DElncRNAs, we predicted their trans- and cis-target genes based on distinct principles and subsequently intersected these with DEmRNAs profiles to enhance prediction accuracy. Trans-target genes were identified using the following criteria: Trans-target genes were identified based on the following criteria: (1) complementary sequences between DElncRNAs and mRNAs with normalized free energy < −0.1 were identified using LncTar software; (2) the Pearson correlation coefficient between DElncRNAs and mRNAs was calculated and found to be |*r*| > 0.97. Cis-target genes were classified as the DEmRNAs transcribed from regions approximately 100 kb upstream and downstream.

### 2.7 Regulatory networks construction and functional analysis

To establish an effective DElncRNAs-mediated protein–protein interaction (PPI) network, all functionally known protein-coding targets (both trans- and cis-targets) of DElncRNAs were selected. The STRING v11.0 database was utilized to generate interactions with the following criteria: a minimum interaction score of ≥0.4 and interactions involving at least one protein. The PPI network was visualized using Cytoscape v3.7.2 software, and the nodes with higher degrees (top 5%) were identified as hub genes through the Cytohubba function. The different modules within the PPI network were delineated using the MCODE function, and the significantly enriched signaling pathways and potential functions were analyzed through KEGG and GO annotation.

### 2.8 Identification of DE-EndMTs

A comprehensive search of the KEGG and GO databases identified a total of 223 genes associated with the Transforming Growth Factor Beta (TGF-β) pathway. Integration with the dbEMT2.0 database (https://bioinfo-minzhao.org/dbemt/index.html) revealed a gene set related to Endothelial-to-Mesenchymal Transition (EndMT), comprising 1,164 genes (see Supplementary Table 2). DEmRNAs were categorized into upregulated (log2FC ≥ 0.5, *p* < 0.05) and downregulated (log2FC ≤ −0.5, *p* < 0.05) groups. DE-EndMTs were defined as the intersection of these DEmRNAs (|log2FC| ≥ 0.5, *p* < 0.05) with the EndMT-related gene set (1,164 genes). Venn diagrams were used to visualize the overlap between the DEmRNAs and the EndMT-related gene set.

### 2.9 Machine learning-based selection of EndMT-related signature genes

To select characteristic genes, three machine learning algorithms were employed: Random Forest, support vector machine recursive feature elimination (SVM-RFE), and least absolute shrinkage and selection operator (LASSO) logistic regression. The LASSO model was constructed using the R package “glmnet” to mitigate the risk of overfitting. A 10-fold cross validation method was applied to find the regularization parameter lambda, which gave the minimum mean cross-validated concordance index. The SVM-RFE model, a supervised machine learning method, was implemented using the e1071 package. This algorithm recursively eliminates features while evaluating classification performance via 10-fold cross-validation at each iteration, ultimately retaining the minimal feature subset that maximizes predictive accuracy and mitigates overfitting risks. The Random Forest model was implemented using the “RandomFores” package, which utilizes a decision tree classifier to iteratively evaluate categorical variables, resulting in the generation of highly accurate classification features. Hyperparameter optimization involved out-of-bag (OOB) error analysis, where ntree (number of trees) and mtry (features per split) were systematically tuned. After evaluating mtry across a range of 1–20 and monitoring OOB error stabilization with ntree = 1,000, the optimal configuration (mtry = 5, ntree = 1,000) was selected to maximize generalizability while minimizing overfitting risks (10).

### 2.10 Construction and evaluation of diagnostic model

Using the “rms” package, a nomogram model was developed to predict the occurrence of atherosclerosis by utilizing selected genetic markers. Calibration curves were plotted to evaluate the predictive accuracy of the nomogram. To assess the clinical significance of the model, decision curve analysis and clinical impact curves were generated (11). Additionally, to further evaluate the diagnostic capacity of the selected genes for atherosclerosis, the “pROC” program was employed, and validation was conducted using the datasets GSE43292, GSE28829, and GSE163154.

### 2.11 Processing of scRNA-Seq data

Single-cell transcriptome profiles of human carotid atherosclerotic plaques were obtained from tissue samples of 12 subjects (6 symptomatic) from the Gene Expression Omnibus (GEO) database (accession code GSE253903). For the analysis of the scRNA-seq data, we utilized the “Seurat”package (version 4.1.2). Initially, we excluded cells with more than 6,000 detected genes and those with more than 30% mitochondrial reads for downstream analysis. The SCTransform function, using default parameters, was employed to normalize and scale the feature expression measurements for each cell based on total expression. Initial cell clustering was performed using the FindClusters function with the first 20 principal components (PCs) and the Louvain algorithm at a resolution of 0.6. Non-linear dimensional reduction was executed via the RunUMAP function and visualized through Uniform Manifold Approximation and Projection (UMAP). Marker genes for each cell cluster were identified using the “FindAllMarkers” function with the Wilcoxon rank-sum test, considering only those with |avg_logFC| ≥ 0.10 and *p*_val ≤ 0.05 as marker genes. We also conducted differential expression analysis on ECs clusters using the “FindAllMarkers”function embedded in Seurat (version 4.1.2) to identify informative markers reflecting the plaque state.

### 2.12 Coexpression analysis of De-EndMTs and DElncRNAs and construction of the ceRNA network

To determine the correlation between DE-EndMTs and DElncRNAs, a coexpression analysis was conducted using the Psych R package, focusing on the identified upregulated and downregulated genes. A relevance value exceeding 0.90 and a *p*-value below 0.05 were established as thresholds. The starBase and multiMiR R packages, which are tools for predicting miRNA binding sites, were employed to estimate the miRNAs that may interact with DElncRNAs and DE-EndMTs. Subsequently, an intersection analysis was performed to construct ceRNA networks composed of both upregulated and downregulated lncRNAs. This process involved integrating miRNA/DElncRNAs and miRNA/DE-EndMTs. The results were visualized using Cytoscape 3.7.1. To elucidate the relevant molecular mechanisms further, the cytoHubba R package, utilizing the MCC method, was adopted to identify the top ten hub lncRNAs within the two ceRNA networks.

### 2.13 qRT-PCR validation

Quantitative reverse transcription polymerase chain reaction (qRT-PCR) was performed to validate the expression levels of three lncRNAs and mRNAs within the competing endogenous RNA (ceRNA) networks in patients with coronary artery disease (CAD) and healthy controls. The study received approval from the Medical Ethics Committee of Hubei University of Medicine, and the characteristics of the patients are detailed in Supplementary Table 4. Residual peripheral blood samples were collected from 50 CAD patients in the cardiovascular department and 50 gender- and age-matched healthy individuals from the health check-up center at Taihe Hospital between April 2024 and June 2024. Inclusion criteria and exclusion criteria were the same as previously reported (12).

Total RNA was extracted from serum samples using Trizol reagent (Vazyme, Nanjing, China) according to the manufacturer's instructions. The concentration and quality of RNA were assessed using a microplate reader. For lncRNA analysis, total RNA was reverse transcribed into complementary DNA (cDNA) using the RevertAid First Strand cDNA Synthesis Kit (Thermo Scientific, Waltham, Massachusetts, USA). For mRNA, equal amounts of RNA were reverse transcribed into cDNA using the HiScriptQ RT SuperMix for qPCR (Vazyme, Nanjing, China). Subsequently, cDNA samples were mixed with ChamQ™ SYBR® qPCR Master Mix (Vazyme, Nanjing, China) and amplified on a Bio-Rad CFX96 PCR Thermal Cycler. The housekeeping genes GAPDH and β-actin served as internal controls. Primer sequences are provided in Supplementary Table 4. Primer specificity was validated via melt curve analysis. qRT-PCR reactions were performed in triplicate using RNase-free consumables. No-template controls (NTCs) were included in each run. Pre- and post-PCR workflows were spatially segregated, and UV sterilization was applied to eliminate contamination. The results were calculated using the 2 −ΔΔCt method.

## 3 Results

### 3.1 lncRNA and mRNA transcriptome

**Figure 1:**
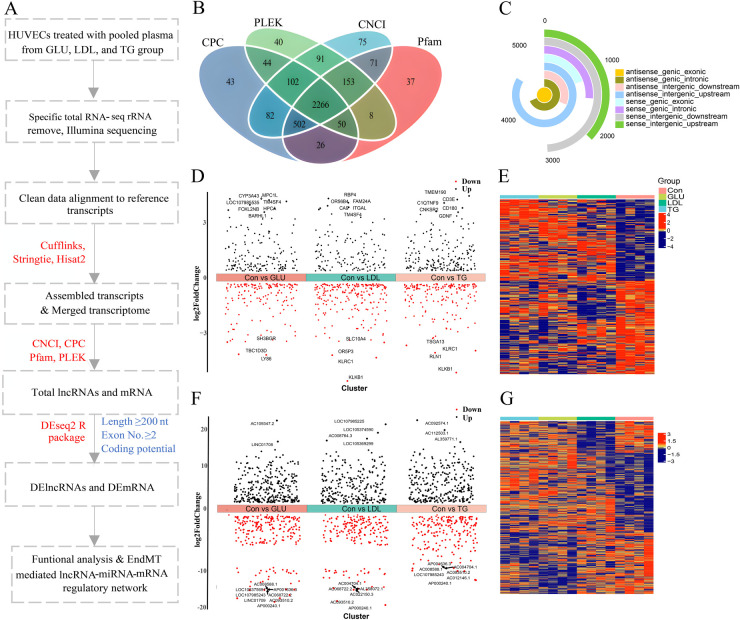
Identification of the differentially expressed RNAs expression profiles in human umbilical vein endothelial cells. **(A)** Flow diagram representing the strategy. HUVECs treated for 72 h with pooled plasma from the Con, GLU, LDL, or TG groups, respectively. whole transcriptome sequencing and bioinformatic analysis was performed. Software used in this study were indicated in red, and the criteria for lncRNAs and DElncRNAs identification were shown in blue. **(B)** Venn diagram showing the lncRNAs identified by four different algorithms (CPC, PLEK, CNCI, and Pfam). **(C)** The pie chart illustrates the number of lncRNAs categorized by their genomic location, with the antisense intergenic upstream category being the most abundant. **(D–G)** Volcano plot showing the expression profiles of differentially expressed mRNAs **(D)** and lncRNA **(F)** in the GLU, LDL, and TG groups compared to the Con group. Differentially expressed mRNAs were identified using the criteria of *p*-value < 0.05 and |Log2 (fold change)| ≥ 0.5, while lncRNAs were identified with *p*-value < 0.05 and |Log2 (fold change)| ≥ 1. Up- and down-regulated genes are labeled in red and black, respectively. Heatmap representing the DEmRNAs **(E)** and DElncRNA **(G)** across the four groups. The color of cells reflects the change degree of DEmRNAs and DElncRNAs expression, blue indicates down-regulated while red indicates up-regulated. Statistical significance was determined using *P*-value < 0.05.

To elucidate the transcriptomic changes in lncRNA and mRNA in HUVECs exposed to pooled serum from the GLU, LDL, TG and Con groups, we employed a high-throughput RNA sequencing strategy (Figure 1A). In brief, we isolated high-quality total RNA from HUVECs to capture their expression profiles and infer the potential functions of lncRNAs and mRNAs using RNA-seq data. This approach generated 229.48 Gb of clean data, with an average of 123,138,624,728 reads per sample, and a Q30 quality score exceeding 95%, ensuring high reliability (Supplementary Table 5). Utilizing a two-iteration mapping method, we aligned transcripts to the annotation database and identified known mRNAs and lncRNAs. Based on our cut-off criteria, we predicted a total of 2,266 novel lncRNAs across the 16 samples analyzed (Figure 1B). At the transcript level, we identified a total of 23,999 lncRNAs, including 5,775 antisense genic exonic, 3,956 antisense genic intronic, 1,826 antisense intergenic downstream, 4,837 antisense intergenic upstream, 1,123 sense genic exonic, 1,510 sense genic intronic, 2,834 sense intergenic downstream, and 2,138 sense intergenic upstream lncRNAs. Notably, the antisense intergenic upstream category was the most abundant (Figure 1C).

To gain further insight into the mRNA and lncRNA responses in HUVECs, we analyzed DEmRNAs and DElncRNAs. In our gene-level expression analysis, we identified a total of 306 DEmRNAs between the control and GLU groups, 335 between the control and LDL groups, and 364 between the control and TG groups, using a significance threshold of *p*-value <0.05 and |log2(fold change)| > 0.5 (Figure 1D). Furthermore, by applying a more stringent threshold of *p*-value < 0.05 and |log2(fold change)| > 1, we identified 523 DElncRNAs in the GLU group (260 upregulated and 263 downregulated), 471 in the LDL group (259 upregulated and 212 downregulated), and 562 in the TG group (293 upregulated and 269 downregulated), compared to the control group (Figure 1F). Heatmaps were utilized to visualize the distribution of differentially expressed mRNAs and lncRNAs (Figures 1E,G).

### 3.2 Enrichment analysis of overlapping DEmRNAs

**Figure 2:**
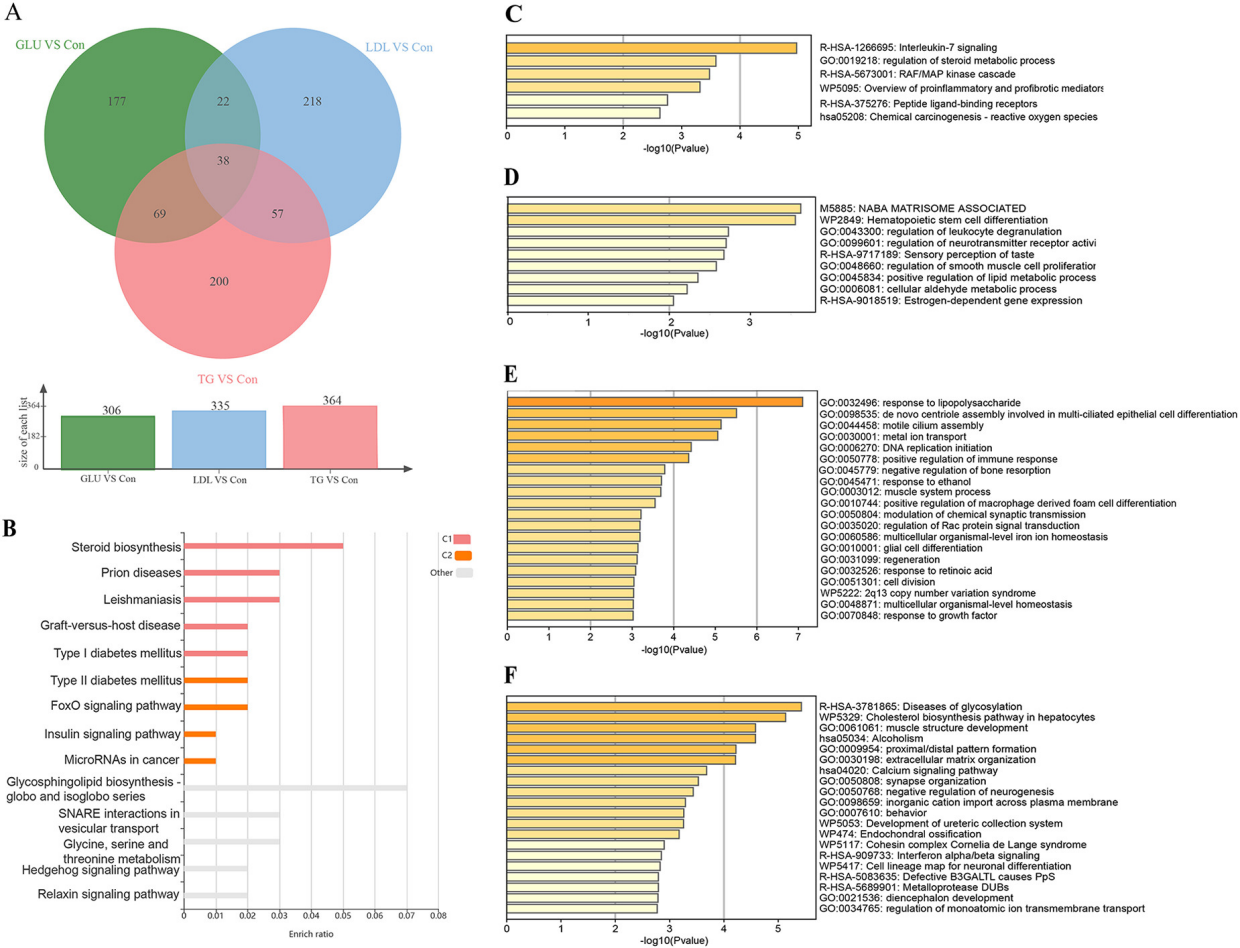
Functional annotation of shared and unique differentially expressed mRNAs (DEGs). **(A)** Three-way Venn diagram showing shared DEGs among the GLU, LDL, and TG groups compared to the Con group. **(B,C)** Enrichment analysis of shared DEGs based on the KOBAS **(B)** and Metascape **(C)** databases, revealing significant enrichment in pro-inflammatory and profibrotic pathways (*P*-value <0.05). (D-F) Enrichment analysis of unique DEGs without overlap among the GLU vs. Con **(D)**, LDL vs. Con **(E)**, and TG vs. Con **(F)** groups based on the Metascape database, highlighting unique biological processes and pathways in each group. Pathway enrichment results were considered significant at a *P*-value threshold of <0.05.

Gene enrichment analysis was performed on the overlapping sets of DEmRNAs to elucidate the shared molecular mechanisms underlying the response of HUVECs to metabolic stressor stimulation. The Venn diagram (Figure 2A) illustrates the distribution of overlapping DEmRNAs across the three groups. KEGG enrichment analysis of the shared differentially expressed mRNAs revealed significant enrichment in microRNAs associated with cancer. Additionally, Metascape analysis indicated enrichment in pro-inflammatory and profibrotic pathways (Figures 2B,C), suggesting a close association of HUVECs with inflammatory and fibrotic processes under these adverse conditions. Furthermore, Metascape was utilized to analyze the exclusive DEmRNAs that exhibited no overlap among the three groups. The results indicated that the GLU group demonstrated significant enrichment in several key biological processes, including the regulation of smooth muscle cell proliferation, regulation of leukocyte degranulation, and positive regulation of lipid metabolic processes (Figure 2D). In the LDL group, the enrichment analysis highlighted critical biological processes, such as the response to lipopolysaccharide, the positive regulation of macrophage-derived foam cell differentiation, the initiation of DNA replication, and metal ion transport, among others (Figure 2E). Furthermore, in the TG group, An interesting unique differential gene enrichment analysis revealed significant activation of the cholesterol biosynthesis pathway in hepatocytes (Figure 2F).

### 3.3 Enrichment analysis of differentially expressed mRNAs

The GSEA was carried out on mRNA expressions across three distinct groups (Supplementary Figures 1A–C). A significant upregulation was witnessed in pathways such as ECM—receptor interaction and the Hedgehog signaling pathway within the GLU group. The main upregulation pathways identified in the LDL group were focused on leukocyte transendothelial migration and the T cell receptor signaling pathway. The TG group showed an upregulation of the Hedgehog signaling pathway, the JAK-STAT signaling pathway, the PI3K-Akt signaling pathway, and the TGF-beta signaling pathway. Further clarification was offered by functional enrichment analysis through GO and KEGG for the DEmRNAs. Significant enrichment was observed in GO terms related to biological processes, including cell adhesion, proliferation, differentiation, and the regulation of immune responses across the three groups. The most positively enriched GO terms related to biological processes are presented in Supplementary Figures 1D–F. Meanwhile, KEGG pathway analysis revealed that the DEmRNAs in the GLU group were highly enriched in pathways such as “Cell adhesion molecules”, “Cytokine-cytokine receptor interaction”, and “Type I diabetes mellitus”. In contrast, the primary KEGG pathways enriched in the LDL and TG groups were predominantly related to inflammation, encompassing pathways such as Lipid and Atherosclerosis, TNF signaling, NF-kappa B signaling, and IL-17 signaling. Notably, the TGF-beta signaling pathway was significantly enriched in the LDL group (Supplementary Figures 1G–I).

### 3.4 Functional assessment of the DElncRNAs

**Figure 3:**
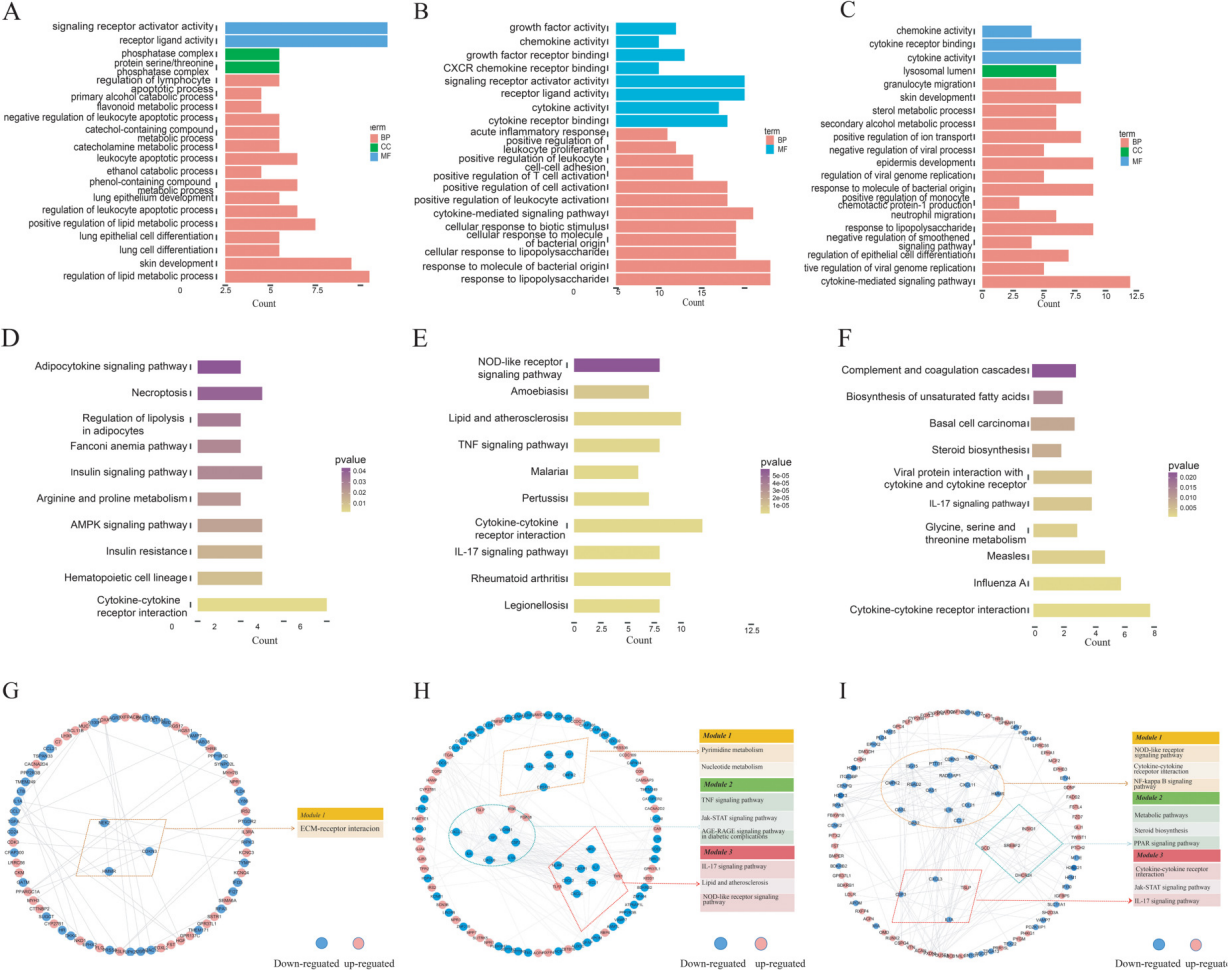
Functional annotation of differentially expressed lncRNA. **(A–C)** KEGG pathway enrichment analyses of DElncRNAs in HUVECs treated with serum from the GLU **(A)**, LDL **(B)**, and TG **(C)** groups compared to the Con group. The top 10 significant KEGG terms were presented, with the color and length of the corresponding column indicating the *p* value and gene number, respectively. (D-F) GO analyses of DElncRNAs in HUVECs treated with serum from the GLU **(D)**, LDL **(E)**, and TG **(F)** groups. The top 20 most enriched GO terms were listed. The columns in red, blue, and green indicate the significantly enriched terms of CC, MF, and BP categories, respectively. **(G–I)** Identification of the DElncRNAs-mediated PPI network in the GLU vs. Con **(G)**, LDL vs. Con **(H)**, and TG vs. Con **(I)** comparisons. The DElncRNAs-mediated PPI network was established based on the targets of DElncRNAs identified in this study. The dotted areas indicate three significant modules (Module I in orange, Module II in green, and Module III in blue). The enriched signaling pathways (*P*-value <0.05) in each module were presented.

To evaluate the potential functions of DElncRNAs in HUVECs, candidate targets were predicted through both trans- and cis-acting regulatory modes. A total of 198, 193, and 220 potential targets were identified across three groups. Based on these targets, GO analyses were conducted, resulting in the identification of significantly enriched GO terms (*p* < 0.05). Notably, DElncRNAs were primarily enriched in GO terms associated with cell growth, metabolism, and differentiation within the GLU group. In contrast, the GO terms enriched in the comparison between the LDL and TG groups were predominantly related to the activation and regulation of the immune system, inflammatory responses, and cell growth and differentiation, as illustrated in Figures 3A–C. Furthermore, KEGG pathway enrichment analyses revealed 10 significantly enriched signaling pathways (*p* < 0.05), including insulin resistance, adipocytokine signaling pathway, and cytokine-cytokine receptor interaction (Figure 3D) in the GLU group. KEGG pathway analysis also demonstrated that the IL-17 signaling pathway and cytokine-cytokine receptor interaction were significantly enriched in the LDL and TG groups (Figures 3E,F). These results indicate that differentially expressed lncRNAs and mRNAs play a crucial role in regulating cellular functions such as metabolism, immune responses, and differentiation in response to metabolic stressors.

### 3.5 DElncRNAs-mediated PPI network construction and module identification

To construct the DElncRNAs-mediated PPI network, we utilized the STRING online database and the Cytoscape v3.7.2 visualization tool, selecting protein-coding targets of DElncRNAs for interaction analysis. As shown in Figure 3H, we identified 74 nodes (34 upregulated and 40 downregulated) and 70 edges in the DElncRNAs-mediated PPI network for the GLU group. Five high-degree nodes (top 5%, average degree ≥ 5) were considered hub genes, including IL1A, MUC1, FOXA1, TGFA, and HGF. Additionally, a significantly enriched module, termed Module I, was identified. KEGG analyses revealed that the primary signaling pathway associated with this module was the ECM-receptor interaction pathway (Figure 5G). Similarly, in the DElncRNAs-mediated PPI networks, we identified 99 nodes and 268 edges in the LDL group, while 99 nodes and 207 edges were identified in the TG group. In the LDL group, IL1B, PXDNL, CDK1, POU5F1, and ISG15 were identified as hub genes, whereas IL6, CXCL8, IL1A, VCM1, and CSF2 were recognized as hub genes in the TG group. Furthermore, three notable enriched modules, designated as Module I, II, and III, were also identified. The signaling pathways primarily associated with these three modules were linked to multiple inflammation pathways, including NF-κB, IL-17, and NOD-like receptor signaling pathways (Figures 5H,I).

### 3.6 Building and verifying machine learning models

**Figure 4:**
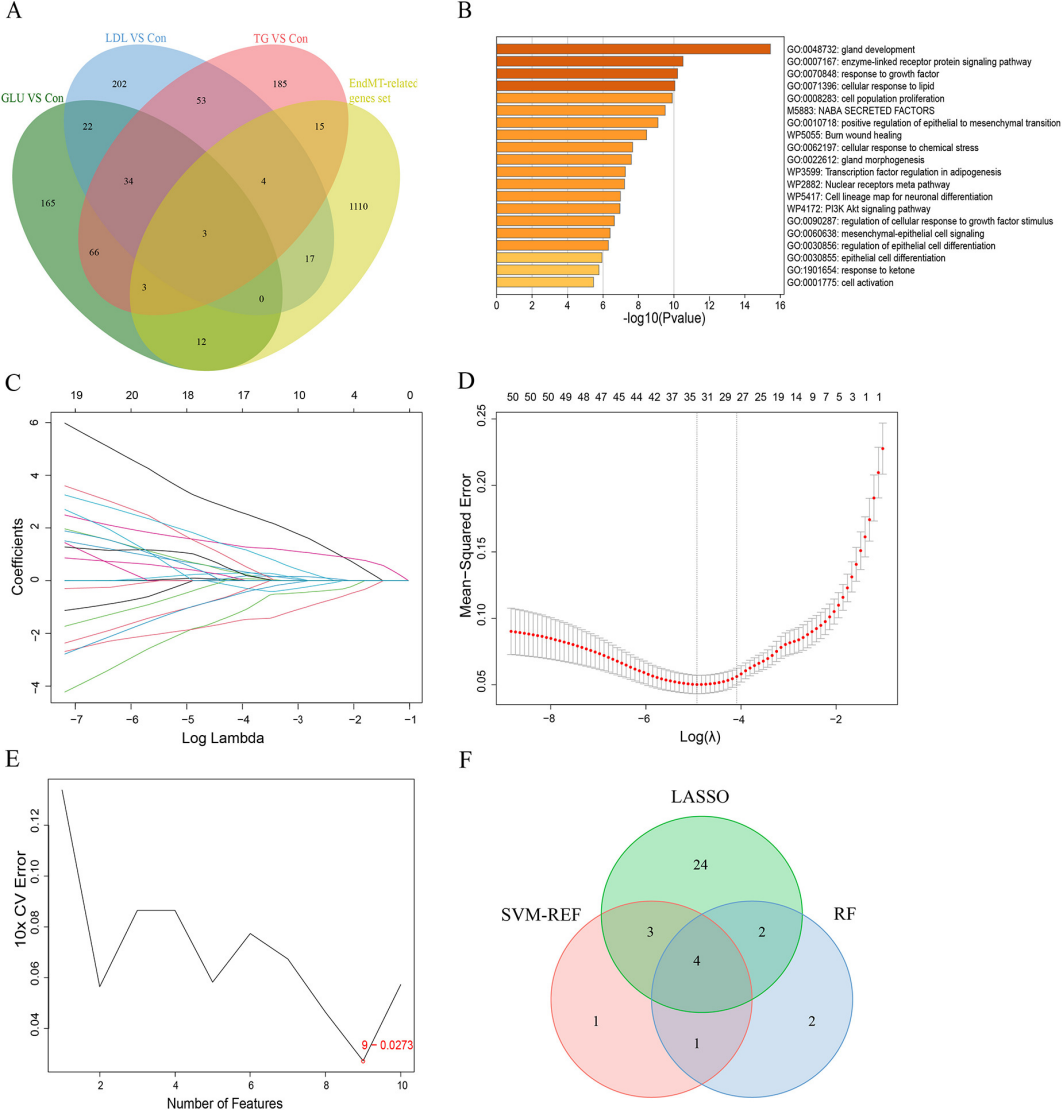
Identification of EndMT-related genes that indicate the presence of a disease. **(A)** Four-way Venn diagram showing the intersection of DEmRNAs from the GLU vs. Con, LDL vs. Con, and TG vs. Con groups with the EndMT-related gene set. **(B)** Enrichment analysis of DE-EndMTs based on the Metascape database. **(C)** 10 cross-validations of the LASSO model's altered parameter selection. Each curve represents a single gene. **(D)** Analysis of LASSO coefficients. The best lambda is where vertical dashed lines are drawn. **(E)** The SVM-RFE algorithm's gene cross-validation error variation curve. **(F)** A Venn diagram illustrates four signature genes related to EndMT that are shared among the LASSO, SVM-RFE and random forest algorithms.

We identified 52 genes associated with EndMT that were differentially expressed across three groups by intersecting differential mRNA expressions with an EndMT-related gene set, as depicted in Figure 4A and Supplementary Table 6. To confirm the association of these genes with EndMT, we conducted a Metascape enrichment analysis. This analysis revealed significant enrichment in four critical domains: epithelial differentiation, regulation of epithelial differentiation, mesenchymal-epithelial signaling, and positive regulation of the epithelial-to-mesenchymal transition (Figure 4B). To investigate the utility of these EndMT-related genes as biomarkers, we utilized GSE100927 as a test dataset and GSE43292, GSE28829, and GSE163154 as validation datasets. We employed three machine learning algorithms: the Least Absolute Shrinkage and Selection Operator (LASSO), Support Vector Machine Recursive Feature Elimination (SVM-RFE), and Random Forest, to evaluate their biomarker potential. Figures 4C–E demonstrate that LASSO regression and SVM-RFE identified a total of 22 and 9 genes, respectively. The Random Forest method utilized gene significance scores to prioritize the genes. The intersection of the top 10 genes with the highest scores from the Random Forest algorithm and the genes obtained through LASSO regression and SVM-RFE resulted in the identification of four genes: ISG15, CD36, HSPB2, and IRS2 (Figure 4F).

### 3.7 Development of nomogram diagram model

**Figure 5:**
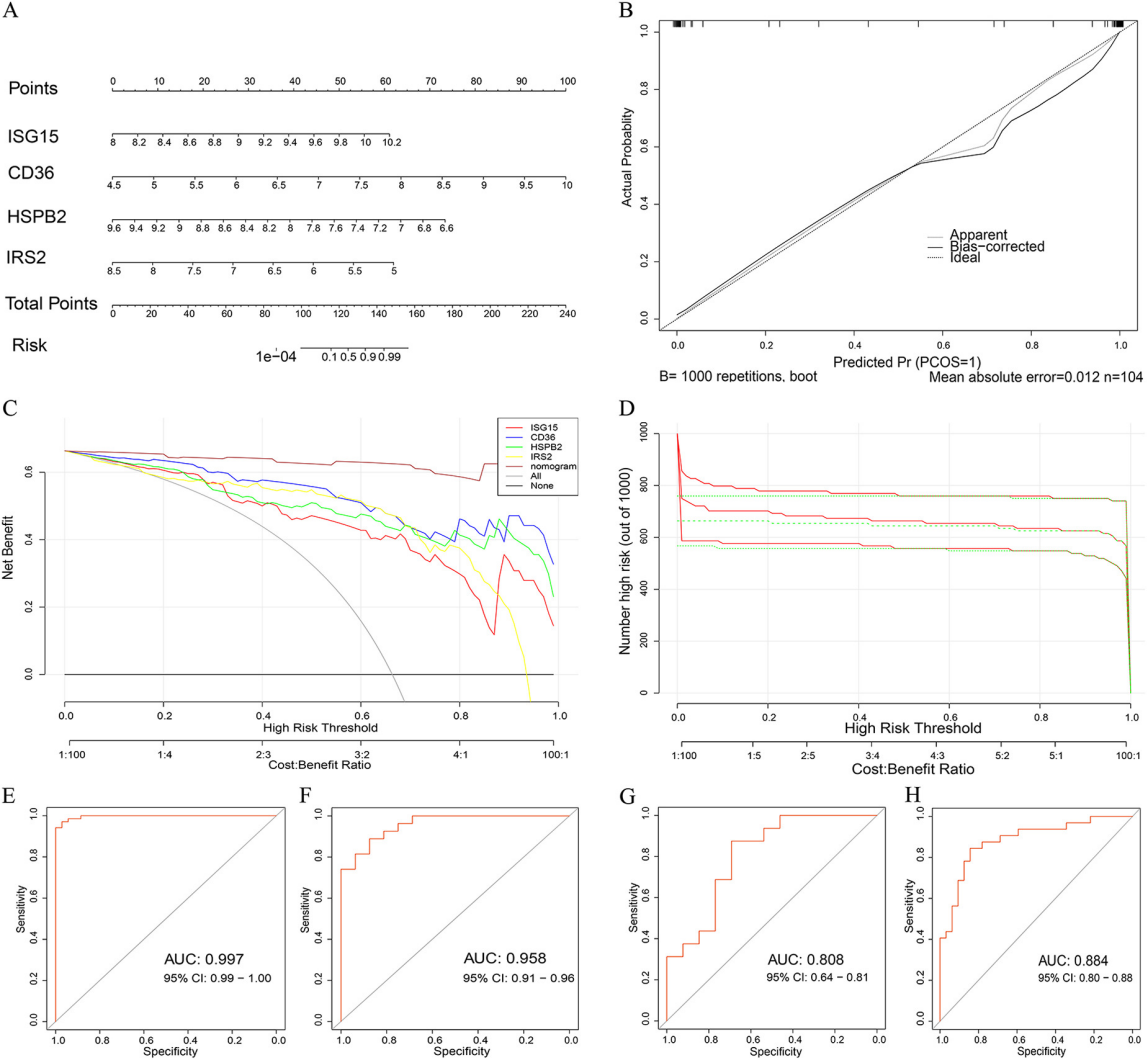
Developing and assessing a nomogram model for the diagnosis of AS. **(A)** Developing a nomogram that utilizes five characteristic genes to forecast the likelihood of AS. **(B)** Calibration curves are used to determine the nomogram's accuracy in making predictions. **(C)** The clinical impact curve determines the clinical significance of the nomogram model. **(D)** The decision curve analysis illustrates the therapeutic benefit of a nomogram. **(E)** The model's ROC curve is shown for the GSE100927 dataset in the training set. **(F)** The validation set GSE163154 dataset displays the ROC curve of the model. **(G)** The model's ROC curve in the GSE28829 dataset. **(H)** The model's ROC curve in the GSE43292 dataset.

Utilizing four key genes identified via machine learning, we developed a nomogram to predict the risk of atherosclerosis development (Figure 5A). According to the clinical impact curve, the nomogram diagram model exhibited robust diagnostic abilities. In addition, we assessed the diagnostic significance of ISG15, CD36, HSPB2, IRS2 and the nomogram diagram model in the GSE100927 dataset by analyzing the area under the ROC curve (Figure 5E). Furthermore, in order to verify the precision of the model, we conducted validation using the GSE43292, GSE28829 and GSE163154 dataset (Figures 5F–H). This indicates that our model not only has high diagnostic value in predicting the occurrence of atherosclerosis, but it is also effective in distinguishing between early and late stages, as well as between IPH and non- IPH patients.

### 3.8 Single-cell sequencing

**Figure 6:**
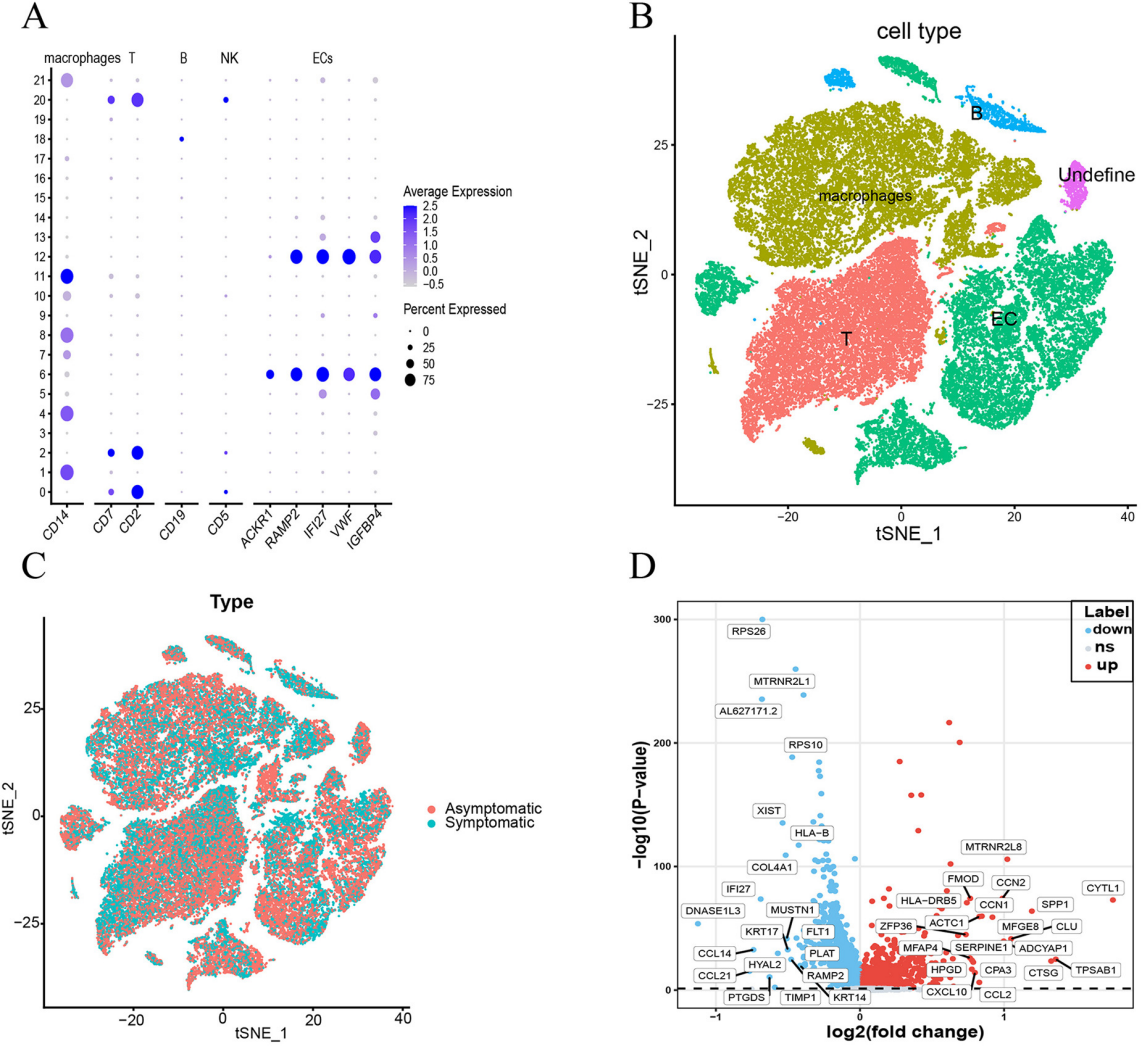
Dimensionality reduction, clustering, and specific markers of different cell types based on single-cell data from the GSE253903 data set. **(A,B)** t-SNE plots of symptomatic and asymptomatic AS patient cells depicting 5 cell types and dot plots of their characteristic genes. **(C)** t-SNE plot illustrating the distribution of cells from symptomatic (blue): and asymptomatic (red): atherosclerosis patients. **(D)** Volcano plots of the most significantly differentially expressed genes in endothelial cells.

To further validate the differential expression of these genes in ECs, we analyzed the single-cell dataset GSE253903. We processed a total of 11,756 cells from 12 samples of six aortic stenosis patients using the Seurat R package. Following quality control and utilizing SingleR and cell taxonomy markers, we annotated 21 subpopulations and identified five distinct cell types (Figures 6A–C and Supplementary Figure 2). To examine gene expression differences in endothelial cells between symptomatic and asymptomatic AS, we conducted a differential analysis on the GSE253903 dataset (Figure 6D). The results demonstrated that the IRS2 and ISG15 genes exhibit significant differential expression in endothelial cells (Supplementary Table 7).

### 3.9 Identification of the EndMT-related ceRNA network

**Figure 7:**
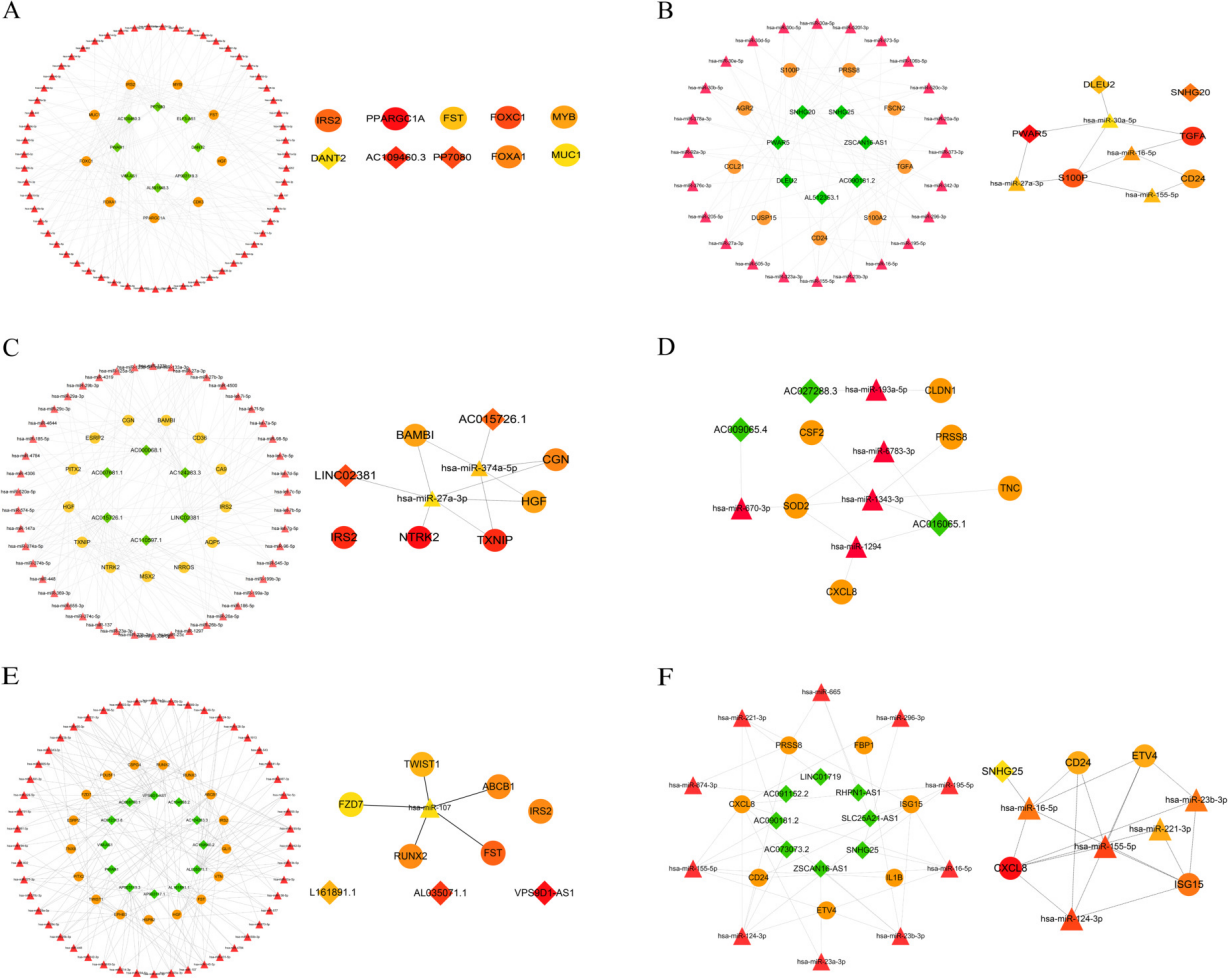
Construction of ceRNA networks related to EndMT. Upregulated lncRNA-associated ceRNA networks in the comparisons of GLU vs. Con **(A)**, LDL vs. Con **(C)**, and TG vs. Con **(E)**. **(B)** Downregulated lncRNA-associated ceRNA networks in the comparisons of GLU vs. Con **(B)**, LDL vs. Con **(D)**, and TG vs. Con **(F)**. Hub nodes of ceRNA in each group were identified by the MCC method (lncRNAs: rhombus, mRNAs: circle, miRNAs: triangle).

Recent studies have demonstrated that lncRNA-mediated ceRNA networks play a pivotal role in a multitude of biological processes, including immunity, embryonic implantation, metabolism, and disease (13–15). To construct ceRNA regulatory networks, we investigated the negative interactions between DElncRNAs and identified EndMT-DEmRNAs. We established three groups of upregulated and downregulated ceRNA networks using miRDB and starBase. Taking the upregulated GLU group as an example, 128 microRNAs (miRNAs) related to EndMT-DElncRNAs and 461 miRNAs associated with DE-EndMTs were identified. The intersection between the two clusters of miRNAs, including 59 miRNAs, was chosen for the following analysis. Simultaneously, 24 intersecting miRNAs from the downregulated group were found among 174 EndMT-DElncRNAs-related miRNAs and 161 DE-EndMT-related miRNAs. Ultimately, based on the intersecting miRNAs, associated EndMT-DElncRNAs and DE-EndMTs, ceRNA networks of the downregulated lncRNAs and upregulated lncRNAs were constructed, as shown in Figures 7A,B, respectively. CytoHubba analysis with the maximal clique centrality (MCC) method was used to identify hub lncRNAs in the two networks, which included, PWAR5, DLEU2, and SNHG20 in the downregulated network and DANT2, AC109460.3, and PP7080 in the upregulated network.

### 3.10 Validation of quantitative real-time polymerase chain reaction

**Figure 8:**
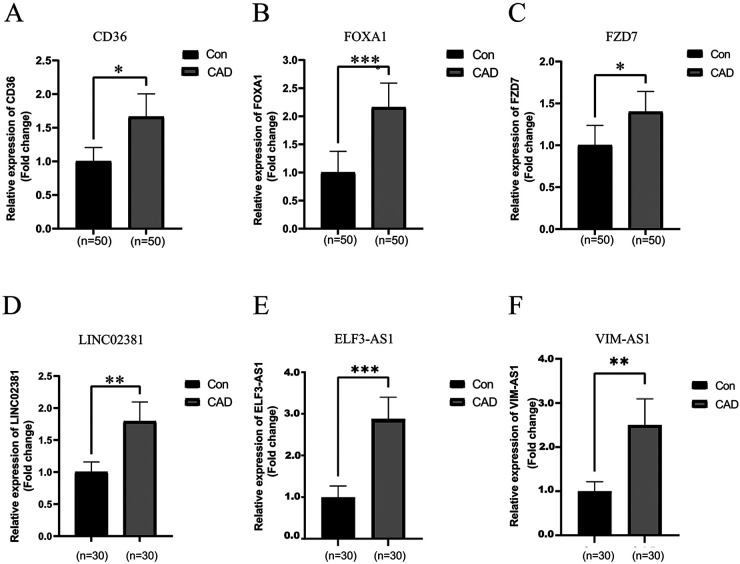
Identification of differentially expressed lncRNAs and mRNAs randomly selected from the ceRNA-network by real-time quantitative polymerase chain reaction. **(A–C)** The relative expression levels of mRNAs in peripheral blood from 50 CAD patients and 50 healthy controls were calculated using the 2 (−*ΔΔ*Ct) method and presented as the mean ± standard error of the mean (SEM). **(D–F)** The relative expression levels of lncRNAs in peripheral blood from 30 CAD patients and 30 healthy controls were determined. The RT-qPCR results represent the mean values of three independent experiments (*n* = 3). *Indicates significance less than 0.05, **indicates significance less than 0.01, ***indicates significance less than 0.001.

To further validate the expression levels of the aforementioned lncRNAs and mRNAs selected from the ceRNA networks, we assessed their expression levels in 50 CAD and 50 healthy peripheral blood samples using qRT‒PCR (Figures 8A–F). The expression level results for CD36, FOXA1, FZD7, VIM-AS1, ELF-AS1, and LINC02381 were consistent with the corresponding alterations observed in the ceRNA network.

## 4 Discussion

ECs exposed to different milieus undergo dynamic phenotypic switching, a critical aspect of endothelial heterogeneity that is essential for maintaining vascular homeostasis, when deregulated, might result in ED. EndMT is a phenotypic conversion process recognized as a hallmark of numerous cardiovascular diseases (16). During this process, the expression of endothelial markers (such as VE-cadherin, CD31), is downregulated, while the expression of mesenchymal markers (such as FSP-1, α-SMA, Vimentin, Fibronectin1) is upregulated (17). Recent studies have confirmed that EndMT is prevalent during atherosclerosis (AS), driving its progression by increasing the deposition of fibronectin (18) and adhesion molecules, as well as altering the balance of collagen and matrix metalloproteinases (19). EndMT is viewed as a critical step for the initiation and progression of atherosclerosis (20). While Notch, Wnt, the Nuclear Factor-kappa B (NF-κB), TNFα, Endothelin-1 (ET-1) and Caveolin-1 (Cav-1) signaling pathways as well as hypoxia, oxidative stress, hyperglycemia, dyslipidemia and shear stress forces represent EndMT-inducing stimuli (21), the downstream signaling pathways of EndMT are not fully characterized. In this study, we employed RNA sequencing to comprehensively analyze the profiles of circRNA, lncRNA, and mRNA in HUVECs under conditions of metabolic stress. Our results indicate that exposure to hyperglycemia and hyperlipidemia *in vitro* leads to the acquisition of a partial mesenchymal-like epigenetic profile in endothelial cells. Based on the expression profiles of DE-EndMTs, we developed an AS model comprising four DE-EndMTs that demonstrated accurate diagnostic performance and the potential to characterize biological features in AS. Furthermore, a ceRNA network targeting the DE-EndMTs were constructed, which enhances our understanding of post-transcriptional regulatory mechanisms and aids in the identification of potential therapeutic targets.

Hyperglycemia and hyperlipidemia induce persistent epigenetic and metabolic changes in ECs, creating a permissive environment for EndMT. It has been described that ECs use metabolites/precursors for epigenetic regulation of their subtype differentiation and maintain crosstalk through metabolites released by other cell types (22, 23). Consistent with this, the enrichment of thiamine and butanoate metabolism was observed in the GLU treatment group. Thiamine plays an important role in glucose metabolism and thiamine deficiency leads to anaerobic metabolism and lactate formation (24). TGF-β, a key inducer of EndMT, is highly expressed in neointimal lesions and critically regulates lipid metabolism by modulating genes involved in fatty acid oxidation and lipid synthesis (25, 26). Yalamanchi et al. demonstrated that lactic acid significantly enhances the activity of TGF-β peptides (TGF-β1, TGF-β2, and TGF-β3), TGF-β receptors (R1, R2, and R3), and TGF-β function, creating a favorable environment for cell transdifferentiation (27). Butyrate is a well-established Histone Deacetylase (HDAC) inhibitor, which is unique among fatty acids (FAs), plays a critical role in epigenetic regulation (28). Aberrant HDAC expression and activity can promote Epithelial-Mesenchymal Transition **(**EMT) and cancer metastasis, while HDAC inhibitors can prevent EMT (29–31). Additionally, Mesenchymal cells derived from EndMT process reprogram their metabolism and show defective FA metabolism (32). Lipid metabolism dysregulation has been closely linked to the TGF-β/Smad signaling pathway (33), which aligns with the observed enrichment of the TGF-β/Smad signaling pathway in the high-fat groups (LDL group and TG group). Zhao et al. observed in a chronic kidney disease (CKD) model that increased expression of extracellular matrix (ECM) components, such as TGF-β1, connective tissue growth factor (CTGF), and type I collagen, was accompanied by disturbances in purine, lipid, and amino acid metabolism (34). Interestingly,predictions of target genes for DElncRNAs revealed that the target genes were primarily enriched in molecular functions related to the remodeling of ECM components and cell adhesion which serve as important extracellular clues of EndMT (35). Those findings collectively suggests that metabolic stress triggered the process of EndMT via remodeling the epigenetic landscape of endothelial cells and activating the fibrotic and inflammatory signaling pathways.

EndMT is a process that is classified as a specialized form of EMT, in which endothelial cells lose their endothelial characteristics and gain a mesenchymal phenotype (36). To identify genes associated with EndMT activation under metabolic stress, we established an EMT dataset based on a search of the KEGG and GO databases, which identified 223 genes linked to the TGF-β pathway. Further integration with the dbEMT2.0 database revealed a total of 1,164 EndMT-related genes, among which 54 were differentially expressed. Previous studies have proposed that EndMT markers may hold potential for disease staging and prognosis prediction (37). For instance, The PDAC tissues with positive EndoMT index were significantly correlated with T4-staging and showed positive for M2-macrophage index (38). By utilizing machine learning algorithms, we established a diagnostic model based on four differentially expressed genes (CD36, IRS2, ISG15, and HSPB2). Cluster of Differentiation 36 (CD36), which was widely expressed on the surface of endothelial cells in the aorta (39), binds with TGF-β1 to activate the fibrosis process (40). Inhibition of CD36 has been shown to suppress EMT and block the Wnt/β-catenin and TGF-β signaling pathways (41). Insulin receptor substrates 2 (IRS2) is the primary isoform of insulin receptor substrate expressed in ECs (42), dysregulation of IRS1/IRS2 contributes to the metabolic disorder, obesity and diabetes (43, 44). IRS proteins can undergo Ser/Thr phosphorylation induced by TGF-β1 (45), a modification that may play a role in regulating downstream processes of the TGF-β1 signaling pathway. Additionally, siRNA-mediated reduction of IRS2 expression markedly increases basal levels of E-cadherin mRNA and protein in kidney epithelial cells (46), indicating that IRS2 may influence EMT through the modulation of E-cadherin expression. ISG15 (interferon-stimulated gene 15) is a ubiquitin-like protein with chemotactic function that recruits neutrophils in the sites of inflammation (47). The deficiency of ISG15 can lead to systemic type I interferon-mediated inflammation in humans (48). *In vitro* and *in vivo* experiments showed that upregulation of ISG15 inhibited EMT in lung adenocarcinoma (49). Heat shock protein family B member 2 (HSPB2) is a novel and unique member of the small heat shock proteins (HSP) family, mainly expressed in skeletal and heart muscles (50, 51). HSPB2 could regulate glucose metabolism, Warburg effect, and ROS level by affecting metabolic genes, including the synthesis of hexokinase II (HK2) and cytochrome c oxidase 2 (SCO2) (52). HK2 is known to be a key metabolic enzyme by promoting glucose uptake in cells and facilitating the Metabolic reprogramming (53). In mouse models of breast cancer metastasis, HK2 deficiency decreases SNAIL protein levels and inhibits SNAIL-mediated epithelial mesenchymal transition and metastasis (54). Furthermore, recent publications have suggested that ECs directly contribute to vascular calcification through EndMT and the extent of EndMT is correlated with plaque instability (18, 19, 55, 56). Tom Alsaigh et al. conducted single-cell analyses on calcified atherosclerotic core (AC) plaques and patient-matched proximal adjacent (PA) portions of carotid artery tissue, identifying differential expression of CD36, IRS2, ISG15, and HSPB2 in ECs. Further single-cell analysis of the GSE253903 dataset reveals that ISG15 and IRS2 are significantly upregulated in endothelial cells of symptomatic arteries. Collectively, although alternative explanations for the observed gene expression changes, such as general inflammatory responses rather than EndMT-specific effects, cannot be ruled out, the evidence from previous studies suggests that the four differentially expressed genes may play a role in EndMT, warranting further investigation.

Recently, the role of noncoding RNAs, including lncRNA, miRNA, and circular RNA, has been validated in various processes related to EndMT and endothelial dysfunction. For instance, lncRNA MALAT1 modulated TGF-β1-induced EndMT by the down-regulation of miR-145 in neointimal hyperplasia (57). LncRNA H19 overexpression prevented high-glucose-induced EndMT via a TGF-β1-dependent but Smad-independent pathway, regulated through the ERK1/2 MAPK pathway (58). Furthermore, the proposition and validation of the ceRNA hypothesis have provided additional insights into potential mechanisms underlying EndMT. Consequently, a ceRNA regulatory network targeting these 52 genes was constructed in our study, offering a new reference for mechanistic understanding and targeted therapy.

Upregulated Hub lncRNAs (DANT2, AC109460.3, PP7080, LINC02381, AC015726.1, AL161891.1, AL035071.1, and VPS9D1-AS1) and downregulated Hub lncRNAs (SNHG20, DLEU2, PWAR5, AC009065.4, AC027288.3, AC016065.1, and SNHG25) were selected from the ceRNA networks. Existing evidence indicates that lncRNA DLEU2 influences biological processes such as EMT and cancer stem cell (CSC) enrichment in breast cancer through the DLEU2/ROR1 axis (59). Others studies have shown that the silencing of DLEU2 inhibited the TGF-β1-induced proliferation, migration and EMT (60). VPS9D1-AS1, has been demonstrated to be overexpressed in various cancer types and identified as a target of Wnt/c-Myc signaling (61). In endometrial cancer, VPS9D1-AS1 promotes tumor progression by acting as a molecular sponge for miR-377-3p, thereby upregulating SGK1 expression and enhancing cell proliferation, invasion, and EMT (62). SNHG20, a newly identified lncRNA, has been found to participate in the process of vasculogenic mimicry (63) and was associated with pulmonary fibrosis through the microRNA 490-3p (miR-490-3p)/TGFBR1 axis (64). LINC02381,was reported to affect ox-LDL-induced endothelial cell injury through the miR-491-5p/transcription factor 7 axis (65). TCF7, a key factor in the Wnt/β-catenin signaling pathway (66), promotes EndMT under ox-LDL conditions. Accordingly, the hub lncRNAs selected in the present study are likely associated with EndMT through various pathways, warranting further *in vivo* or *in vitro* investigations. Moreover, in order to verify the application value of our established ceRNA network, we randomly selected three lncRNAs and their corresponding target mRNAs for verification. Up-regulation or down-regulation of genes in serum samples from patients with atherosclerosis are consistent with the results of ceRNA.

Admittedly, there are several limitations to our study. Firstly, the establishment and validation of the EndMT model were performed on public datasets with small samples; a large-sample validation and optimal cutoff determination are required before clinical translation. Secondly, expression validation cohort was relatively small, It would have a lot more value if a larger sample size were concluded; furthermore, the recruitment, activation, and regulatory roles of these genes in ECs as well as the ceRNA regulatory network require validation in further *in vitro* and *in vivo* experiments.

Despite these limitations, our study offers valuable preliminary insights into the role of EndMT-associated genes in ED. We hope that these findings will lay the groundwork for the future clinical application of these markers in the diagnosis and prognosis of AS and other cardiovascular diseases. In our future work, we plan to validate the regulatory mechanisms of the identified genes in EndMT through gene knockdown and overexpression studies, using animal models for *in vivo* verification. Additionally, we aim to collect more clinical samples to assess the diagnostic potential of these biomarkers across different stages of cardiovascular disease.

## 5 Conclusion

In conclusion, this study utilized pooled human serum to more effectively replicate the endothelial microenvironment associated with metabolic-induced endothelial dysfunction, with a particular focus on biological pathways and changes in gene expression. We identified a novel AS model based on the expression profiles of four DE-EndMTs, which demonstrated significant diagnostic potential and the capability to characterize biological features associated with AS. Furthermore, we constructed a ceRNA network targeting these DE-EndMTs, which elucidates the post-transcriptional regulatory mechanisms involved in ECs and provides insights into potential therapeutic targets. While our findings offer valuable insights into the molecular foundations of ED, further experimental validation is necessary to confirm these results and to explore the therapeutic implications in greater depth.

## Data Availability

The original contributions presented in the study are publicly available. This data can be found here: PRJNA1259178 (https://www.ncbi.nlm.nih.gov/bioproject/PRJNA1259178).
