# Supplementary material for: Characterizing hub biomarkers for metabolic-induced endothelial dysfunction and unveiling their regulatory roles in EndMT through RNA sequencing and machine learning approaches
Source: Front Cardiovasc Med. 2025 May 15;12:1585030. doi: 10.3389/fcvm.2025.1585030 (PMC12119472; doi:10.3389/fcvm.2025.1585030)
Supplement: Supplementary file 1 [file Datasheet1.zip › Supplementary Material/Supplementary Table 1.pdf]

Supplementary Table1 Baseline Biochemical Characteristics of Participants Undergoing Routine Health Check-ups and Matched Healthy Controls

Parameters	Cohort 1: GLU(n =20)	Cohort 2: LDL(n =20)	Cohort 3: TG(n =20)	Cohort 4: Con(n =20)
Age(years)	56.05 ± 9.59	55.05 ± 8.53	55.75±7.07	56.8 ± 10.45
Male	10	7	9	9
TC(mmol/L)	4.0 ± 0.58	6.255 (5.9575, 6.7225)	4.48 ± 0.68	4.60 ± 0.54
TG(mmol/L)	1.19 ± 0.38	1.35 ± 0.26	3.725 （3.0125, 5.08）	1.15 ± 0.35
HDL-C(mmol/L)	1.6 ± 0.62	1.365 (1.2675, 1.5100)	0.99 （0.89, 1.24）	1.4950 （1.2675，2.1075）
LDL-C(mmol/L)	1.77 ± 0.66	3.465 (3.3275, 3.6425)	2.13 ± 0.60	1.67 ± 0.48
Glucose(mmol/L)	11.97 ± 2.09	5.45 ± 0.36	5.33（4.58, 5.75）	5.40 ± 0.24
ALT(U/L)	17.550 (13.300, 23.875)	14.700 (11.375, 17.725)	16.700 (15.375, 21.400)	14.650 (10.975, 23.600)
AST(U/L)	21.800 (18.950, 23.950)	21.43 ± 4.81	23.07 ± 6.35	23.49 ± 7.39
GGT(U/L)	21.200 (15.975, 25.825)	17.600 (12.875, 26.675)	28.50 ± 10.14	20.300 (13.750, 34.350)
ALP(U/L)	81.56 ± 14.32	76.26 ± 21.98	74.54 ± 15.29	76.07 ± 18.38
hs-CRP(mg/L)	1.550 (0.915, 3.515)	0.880 (0.623, 2.160)	1.120 (0.927, 2.810)	0.740 (0.315, 1.452)
WBC(G/L)	5.790 (5.197, 6.275)	5.84 ± 1.42	5.74 ± 1.55	5.25 ± 0.91
ANC(G/L)	3.535 (2.853, 4.053)	2.920 (2.558, 4.470)	3.20 ± 0.93	2.99 ± 0.80
RBC(T/L)	4.715 (4.242, 5.078)	4.515 (4.285, 4.912)	4.47 ± 0.45	4.625 (4.328, 4.982)
Hb(g/L)	145.45 ± 17.78	138.75 ± 19.45	137.20 ± 16.99	143.000 (133.250, 155.250)
MCV(fL)	92.850 (90.500, 95.050)	91.39 ± 5.09	92.22 ± 4.83	92.58 ± 3.78
PLT(G/L)	180.500 (153.250, 246.000)	220.70 ± 57.68	199.85 ± 50.82	217.75 ± 70.30

*Continuous variables are presented as mean ± standard deviation (SD) for normally distributed data or median (interquartile range, IQR) for non-normally distributed data, as determined by Shapiro-Wilk normality tests ($\alpha = 0.05$). TC: Total Cholesterol; TG: Triglyceride; HDL-C: High-Density Lipoprotein Cholesterol; LDL-C: Low-Density Lipoprotein Cholesterol; Glucose: Glucose; ALT: Alanine Aminotransferase; AST: Aspartate Aminotransferase; GGT: Gamma-Glutamyl Transferase; ALP: Alkaline Phosphatase; hs-CRP: High-Sensitivity C-Reactive Protein; WBC: White Blood Cells; ANC: Absolute Neutrophil Count; RBC: Red Blood Cells; Hb: Hemoglobin; MCV: Mean Corpuscular Volume; PLT: Platelet.
