# Supplementary material for: Characterizing hub biomarkers for metabolic-induced endothelial dysfunction and unveiling their regulatory roles in EndMT through RNA sequencing and machine learning approaches
Source: Front Cardiovasc Med. 2025 May 15;12:1585030. doi: 10.3389/fcvm.2025.1585030 (PMC12119472; doi:10.3389/fcvm.2025.1585030)
Supplement: Supplementary file 1 [file Datasheet1.zip › Supplementary Material/Supplementary Table 3.pdf]

Supplementary Table 3 Subject baseline data sheet

Parameters	Control group (*n* = 50)	CAD group (*n* = 50)	*P* Value
Age(years)[1]	61 (57-68)	62 (56-69)	0.417
Male[2]	32	28	0.586
Diabetes[2]	5	10	<0.001
Hyperlipidemia[2]	0	6	0.02
TC (mmol/L)[1]	4.34 (3.75–5.02)	3.90 (3.19–4.57)	0.04
TG (mmol/L)[1]	1.15 (0.86–1.34)	1.40 (0.91–1.86)	0.25
HDL-C (mmol/L)[1]	1.33 ± 0.18	1.16 (1.00, 1.38)	0.04
LDL-C (mmol/L)[1]	2.21 ± 0.51	2.45 ± 0.69	0.32
Glucose (mmol/L)[1]	4.93 (4.49–5.57)	6.40 (5.25–8.58)	0.01

* [1]Measurement data that did not conform to normal distribution were tested by Mann-Whitney U test, and the results were expressed as the median and interquartile intervals. [2]Chi-square test was used for categorical variables.
