# Supplementary material for: Characterizing hub biomarkers for metabolic-induced endothelial dysfunction and unveiling their regulatory roles in EndMT through RNA sequencing and machine learning approaches
Source: Front Cardiovasc Med. 2025 May 15;12:1585030. doi: 10.3389/fcvm.2025.1585030 (PMC12119472; doi:10.3389/fcvm.2025.1585030)
Supplement: Supplementary file 1 [file Datasheet1.zip › Supplementary Material/Supplementary Table 4.pdf]

Supplementary Table 4 Sequences of the primers used in the qRT-PCR experiments

**Primer name**	**Sequence 5'-3'**
GAPDH Forward	GAAGGTGAAGGTCGGAGTC
GAPDH Reverse	GAAGATGGTGATGGGATTTC
β-actin Forward	AAACTGGAACGGTGAAGGTG
β-actin Reverse	AGAGAAGTGGGGTGGCTTTT
CD36 Forward	AAGCCAGGTATTGCAGTTCTTT
CD36 Reverse	GCATTTGCTGATGTCTAGCACA
FZD7 Forward	GTGCCAACGGCCTGATGTA
FZD7 Reverse	AGGTGAGAACGGTAAAGAGCG
FOXA1 Forward	GCAATACTCGCCTTACGGCT
FOXA1 Reverse	TACACACCTTGGTAGTACGCC
LINC02381 Forward	CTGATGGCCACTCACGCTAT
LINC02381 Reverse	GATCCGGAGGGAGAGCATTC
VIM-AS1 Forward	CCATGTGTGCGATTCACAAGCCTT
VIM-AS1 Reverse	TGATGCTGATGCTACAGGTCTGAGTA
ELF3-AS1 Forward	TGAAGTCATCACGAACCGC
ELF3-AS1 Reverse	GGAGCCCCAAGTTAATGCG
