# Supplementary material for: Characterizing hub biomarkers for metabolic-induced endothelial dysfunction and unveiling their regulatory roles in EndMT through RNA sequencing and machine learning approaches
Source: Front Cardiovasc Med. 2025 May 15;12:1585030. doi: 10.3389/fcvm.2025.1585030 (PMC12119472; doi:10.3389/fcvm.2025.1585030)
Supplement: Supplementary file 1 [file Datasheet1.zip › Supplementary Material/Supplementary Table 5.pdf]

Supplementary Table 5 Summary of data from RNA sequencing

Group	Sample	Raw reads	Clean reads	Raw bases(G)	Clean bases(G)	ValidBases (%)	Q30 (%)	GC content(%)
Con	Con-1	89,160,000	88,670,000	13.37	13.14	98.22	95.43	51.74
	Con-2	98,370,000	97,770,000	14.75	14.48	98.16	95.44	53.07
	Con-3	112,040,000	111,460,000	16.81	16.55	98.47	95.56	50.69
	Con-4	100,900,000	100,320,000	15.14	14.89	98.38	95.67	56.25
Glu	Glu-1	85,860,000	85,350,000	12.88	12.64	98.18	95.3	52.38
	Glu-2	110,470,000	109,870,000	16.57	16.34	98.63	95.52	53.67
	Glu-3	109,970,000	109,360,000	16.5	16.23	98.39	95.61	52.46
	Glu-4	100,470,000	99,860,000	15.07	14.79	98.13	95	50.12
LDL	LDL-1	86,360,000	85,870,000	12.95	12.72	98.23	95.29	52.18
	LDL-2	101,090,000	100,520,000	15.16	14.98	98.78	95.47	55.42
	LDL-3	115,810,000	115,200,000	17.37	17.06	98.23	95.56	51.09
	LDL-4	107,240,000	106,510,000	16.09	15.77	98.07	95.09	56.86
TG	TG-1	108,630,000	108,030,000	16.29	16.05	98.52	95.09	51.95
	TG-2	104,930,000	104,330,000	15.74	15.43	98.06	95.35	53.67
	TG-3	102,970,000	102,400,000	15.44	15.2	98.43	95.5	51.96
	TG-4	108,710,000	107,870,000	16.31	15.91	97.6	95.01	52.2
