# Supplementary figures and images for: Characterizing hub biomarkers for metabolic-induced endothelial dysfunction and unveiling their regulatory roles in EndMT through RNA sequencing and machine learning approaches
Source: Front Cardiovasc Med. 2025 May 15;12:1585030. doi: 10.3389/fcvm.2025.1585030 (PMC12119472; doi:10.3389/fcvm.2025.1585030)

A

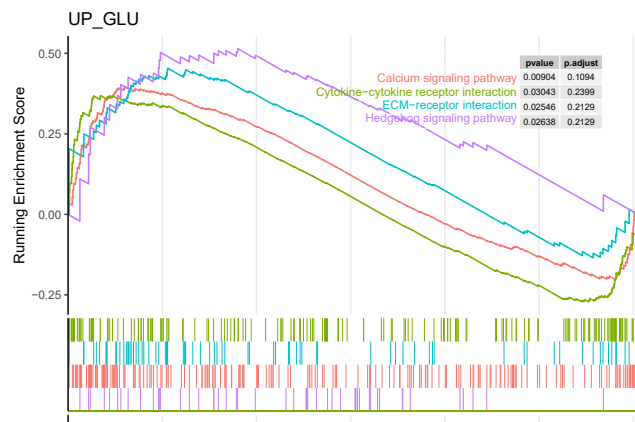

B

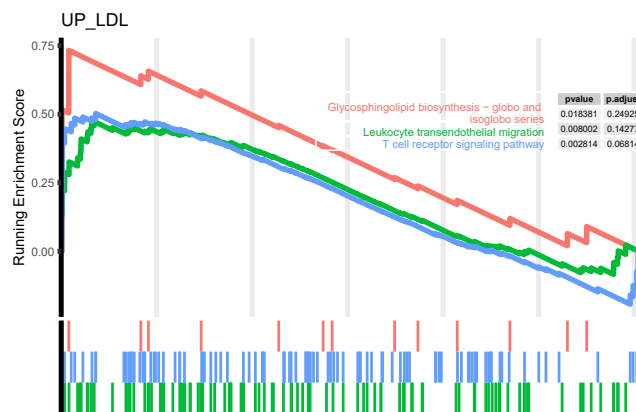

C

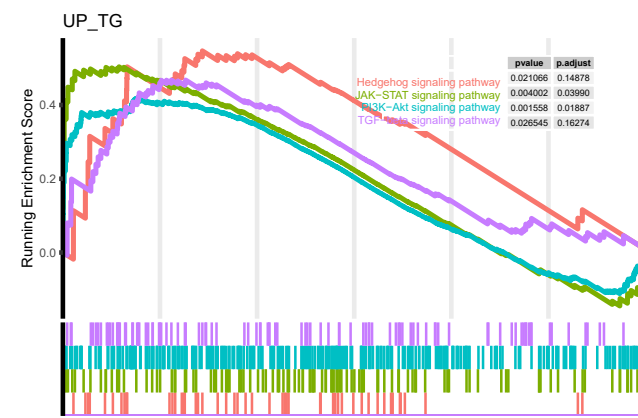

D

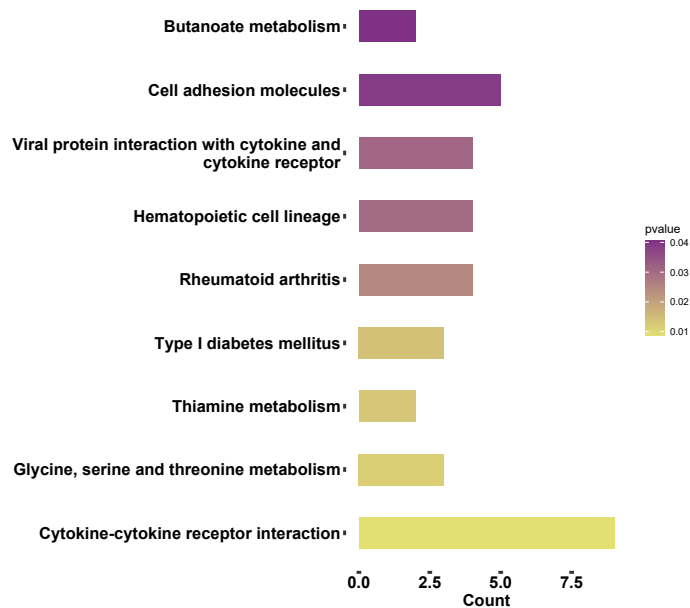

E

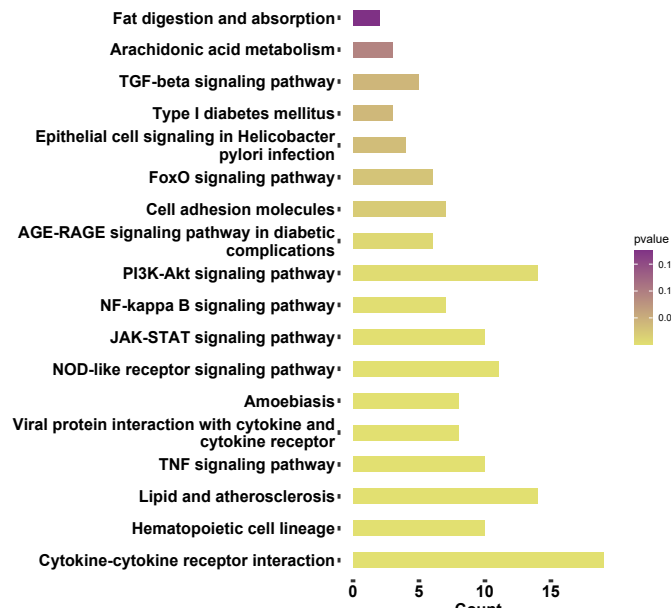

F

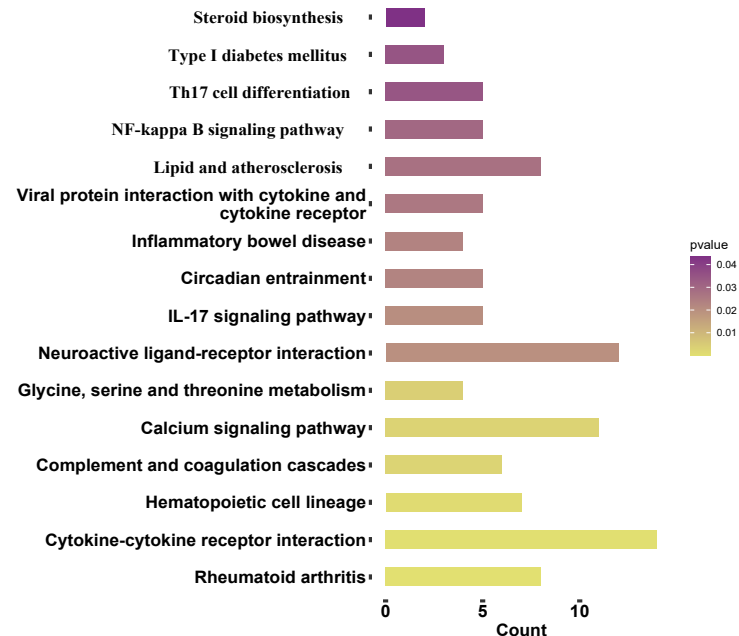

G

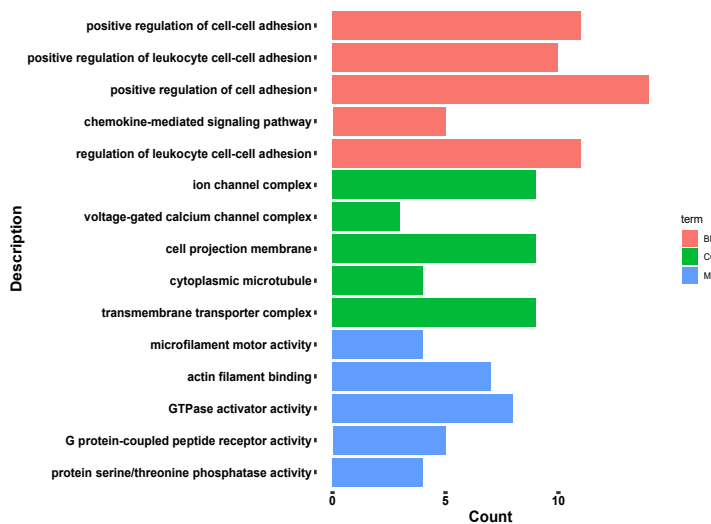

H

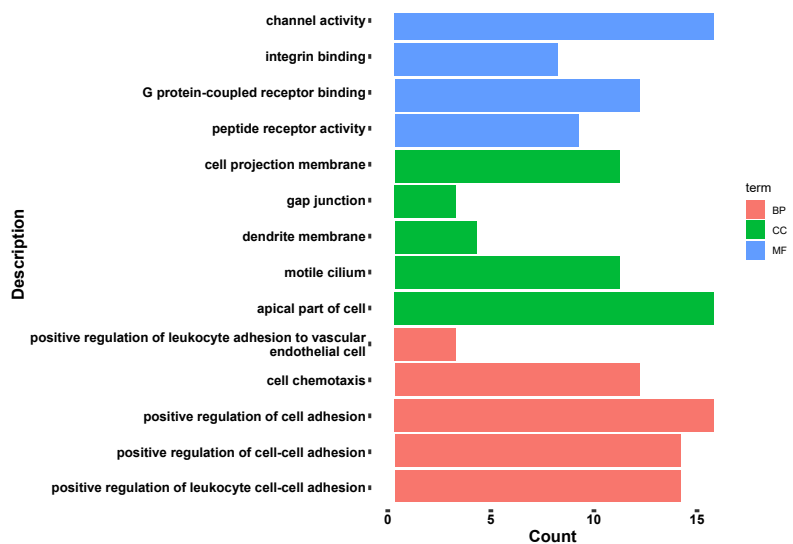

I

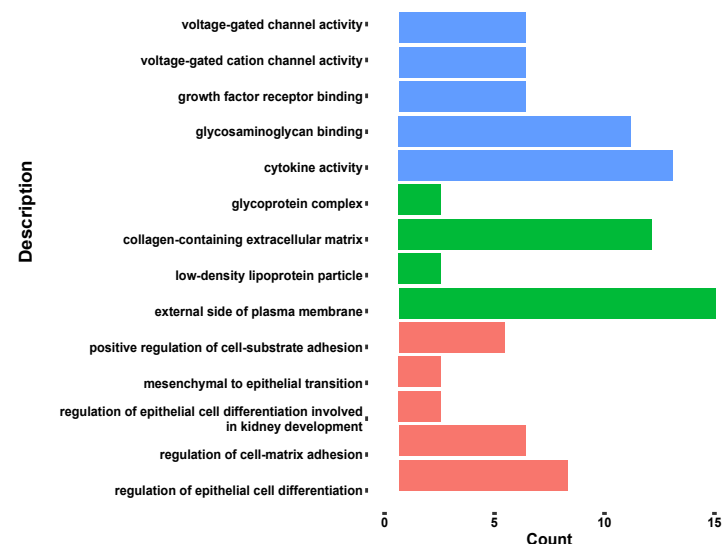

Supplement: Supplementary file 1 [file Datasheet1.zip › Supplementary Material/Supplementary fig.1.pdf]
